# Advancing engagement and capacity for rural cancer control: a mixed-methods case study of a Community-Academic Advisory Board in the Appalachia region of Southwest Virginia

**DOI:** 10.1186/s40900-021-00285-y

**Published:** 2021-06-22

**Authors:** Jamie M. Zoellner, Kathleen J. Porter, Donna-Jean P. Brock, Emma Mc Kim Mitchell, Howard Chapman, Deborah Clarkston, Wendy Cohn, Lindsay Hauser, Dianne W. Morris, Sarah Y. Ramey, Brenna Robinson, Scott Schriefer, Noelle Voges, Kara P. Wiseman

**Affiliations:** 1 Department of Public Health Sciences, School of Medicine, University of Virginia, 16 E. Main St, Christiansburg, VA 24073 USA; 2 School of Nursing, University of Virginia, PO Box 800782, Charlottesville, VA 22908 USA; 3 Tri-Area Community Health, P.O. Box 9, Laurel Fork, VA 24352 USA; 4 Mountain Empire Community College Nursing Program, Phillips-Taylor Hall Rm 136, 3441 Mountain Empire Rd, Big Stone Gap, VA 24219 USA; 5 Department of Public Health Sciences, University of Virginia, School of Medicine, P.O. Box 800717, Charlottesville, VA 22908 USA; 6 Office of Outreach and Engagement, University of Virginia Cancer Center, Box 800334, Charlottesville, VA 22908 USA; 7 Mountain Laurel Cancer Support and Resource Center, Mountain Empire Older Citizens, Inc., P.O. Box 888, Big Stone Gap, VA 24219 USA; 8 Clinch Valley Medical Center, 6801 Gov. G. C. Peery Hwy., Richlands, Virginia, 24641 USA; 9 Kingsport, TN USA; 10 Office of Community Outreach & Engagement, University of Virginia Cancer Center, P.O. Box 800334, Charlottesville, VA 22903 USA

**Keywords:** Cancer, Community-based participatory research, Community capacity, Mixed-methods, Medically underserved area, Healthcare coalitions

## Abstract

**Background:**

The objectives are to: 1) describe engagement processes used to prioritize and address regional comprehensive cancer control needs among a Community-Academic Advisory Board (CAB) in the medically-underserved, rural Appalachian region, and 2) detail longitudinal CAB evaluation findings.

**Methods:**

This three-year case study (2017–2020) used a convergent parallel, mixed-methods design. The approach was guided by community-based participatory research (CBPR) principles, the Comprehensive Participatory Planning and Evaluation process, and *Nine Habits of Successful Comprehensive Cancer Control Coalitions.* Meeting artifacts were tracked and evaluated. CAB members completed quantitative surveys at three time points and semi-structured interviews at two time points. Quantitative data were analyzed using analysis of variance tests. Interviews were audio recorded, transcribed, and analyzed via an inductive-deductive process.

**Results:**

Through 13 meetings, Prevention and Early Detection Action Teams created causal models and prioritized four cancer control needs: human papillomavirus vaccination, tobacco control, colorectal cancer screening, and lung cancer screening. These sub-groups also began advancing into planning and intervention proposal development phases. As rated by 49 involved CAB members, all habits significantly improved from Time 1 to Time 2 (i.e., communication, priority work plans, roles/accountability, shared decision making, value-added collaboration, empowered leadership, diversified funding, trust, satisfaction; all *p* < .05), and most remained significantly higher at Time 3. CAB members also identified specific challenges (e.g., fully utilizing member expertise), strengths (e.g., diverse membership), and recommendations across habits.

**Conclusion:**

This project’s equity-based CBPR approach used a CPPE process in conjunction with internal evaluation of cancer coalition best practices to advance CAB efforts to address cancer disparities in rural Appalachia. This approach encouraged CAB buy-in and identified key strengths, weaknesses, and opportunities that will lay the foundation for continued involvement in cancer control projects. These engagement processes may serve as a template for similar coalitions in rural, underserved areas.

**Supplementary Information:**

The online version contains supplementary material available at 10.1186/s40900-021-00285-y.

## Plain English summary

There has been notable progress among state and local cancer coalitions in developing and implementing comprehesive cancer control plans. Yet, gaps exist in rural communities and cancer remains a leading cause of death in rural populations. This paper reports on how the involvement of a Community-Academic Advisory Board (CAB) helped to prioritize cancer control and research needs in the medically-underserved, rural Appalachian region. CAB members were asked to participate in meetings and to share their experiences through surveys and interviews. Through this three-year process, four cancer control needs were prioritized: human papillomavirus vaccination, tobacco control, colorectal cancer screening, and lung cancer screening. Also, over the course of the project, CAB members’ experiences improved, including: communication, priority work plans, roles/accountability, shared decision making, value-added collaboration, empowered leadership, diversified funding, trust, satisfaction. During the interviews, CAB members identified specific challenges, strengths, and recommendations. The opportunities and barriers at building and sustaining capacity as well as advancing a community-driven research agenda to address cancer disparities in rural Appalachia is discussed.

**Supplementary Information:**

The online version contains supplementary material available at 10.1186/s40900-021-00285-y.

## Introduction

Comprehensive cancer control (CCC) efforts refer to an integrated partnership approach that fosters collaboration among multiple organizations to prioritize, develop, and implement plans that address cancer burden across the cancer control continuum. As such, demand for patient and stakeholder engagement in research has been emphasized across federally funded calls for proposals and cancer control initiatives [1, 2]. Extensive literature highlights both the importance and complexity of building community partnerships and sustaining research capacity [3, 4]. However, despite ongoing recommendations to engage stakeholders in CCC research efforts, the extent of stakeholder involvement through core research phases varies greatly. Historically, there have been relatively few reports of community stakeholders setting research priorities and generating hypotheses [5, 6]. Practical methods are also needed to advance the role of stakeholders along the engagement spectrum (e.g., inform→ consult→ collaborate→ stakeholder directed) [7]. Likewise, there is a need for processes that actively engage stakeholders in formulating and implementing CCC research agendas.

Community-Based Participatory Research (CBPR) and Comprehensive Participatory Planning and Evaluation (CPPE) processes are two promising approaches to address this gap. Effective CBPR initiatives leverage the collective knowledge, expertise, and resources of community-academic partnerships to develop and execute culturally-relevant and community-prioritized interventions [8, 9]. The CPPE process is a five step, action-oriented approach designed to guide project planning and evaluation [10–12]. Furthermore, the Comprehensive Cancer Control National Partnership has developed the Nine Habits of Successful Comprehensive Cancer Control Coalitions (Nine Habits) to guide development and evaluation of CCC efforts [13, 14]. Relying on these approaches and resources to advance CCC efforts is especially important among rural, health disparate communities, where cancer disparities persist [15–17].

### Cancer disparities context in the rural, Appalachia region of Southwest Virginia

While there has been notable CCC progress among state and local cancer coalitions [18–22], cancer remains a leading cause of death in rural populations, and rural-urban disparities persist [17, 23, 24]. Compared to other US counties, rural Appalachian counties have poorer cancer-related health outcomes across the cancer care continuum and suffer from persistent disparities [25–31]. Notably, southwest Virginia and other Appalachian regions face a higher mortality-incidence ratio than other US areas [28]. Lower cancer incidence rates in southwest Virginia is symptomatic of lack of early screening and detection, whereas, higher mortality rates are indicative of later stage diagnosis and lack of access to treatment and cancer survivorship support programs. Specifically, there are noted breast, cervical, colorectal, and lung cancer disparities [25–31]. Additionally, the region faces high prevalence of cancer related risk factors, including higher rates of obesity and tobacco use and lower rates of cancer screening and physical activity [26, 32].

### History of the Southwest Virginia community-academic advisory board (CAB)

In 2013, the University of Virginia (UVA) formed a regional Community-Academic Advisory Board (CAB) to engage local stakeholders in addressing cancer disparities and access to care in rural, southwest Virginia. The CAB’s mission was to provide guidance to ensure cancer control and prevention programs and research served the best interests of residents living and working in the represented health districts. Initial efforts were supported by UVA’s Cancer Center. However, these efforts were neither structured within CBPR initiatives nor adequately funded to provide dedicated staff and technical assistance for regional coalition development.

From 2013 to 2017, the CAB grew to include over 30 representatives from three health districts representing 13 counties in southwest Virginia. Governed in accordance to by-laws and with three formal meetings per year, the CAB was co-chaired by three volunteer community members, representing each health district. Primary achievements of the CAB during this time included 1) identifying regional needs and opportunities, 2) assisting in coordinating cancer outreach programs, and 3) providing insight on feasibility of research projects and collaborate on initiatives. Building from these accomplishments, in 2017, timing was optimal to elevate local leadership and restructure the CAB to develop, prioritize, and implement a regional cancer control research agenda. This paper describes the CAB’s process of transitioning to a community-driven and action-oriented cancer control research agenda in rural Appalachia and highlights the experience of involved stakeholders.

### Objectives

This engagement project focused on strengthening the infrastructure of the CAB, including advancing the knowledge, competencies, and abilities of CAB members to participate in developing and executing patient-centered CCC research and outreach projects. The objectives of this paper are to 1) describe processes that were used to prioritize regional CCC needs, along with intermediate outcomes and 2) detail longitudinal findings (i.e., challenges, strengths, recommendations) from a capacity evaluation. The goals of this engagement project were to increase knowledge exchange and advance the CAB’s readiness and capacity to prioritize and act on cancer-related disparities in rural Appalachia. In accordance with Guidance for Reporting Involvement of Patients and the Public (GRIPP), this study meets all five criteria for quality, consistency, and transparency in patient and public involvement as outlined on the GRIPP2 short form checklist [33]**.**

## Methods

This three-year case study (2017–2020) and process evaluation describes CAB advancement using a convergent parallel mixed-methods design [34, 35]. The approach and evaluation were guided by CBPR principles [8, 9], the CPPE process [10–12], the Nine Habits [13, 14], and coalition evaluation literature [36]. The UVA Intuitional Review Board approved this project. CAB members provided signed informed consent before participating in the evaluation.

### CAB membership

**Fig. 1:**
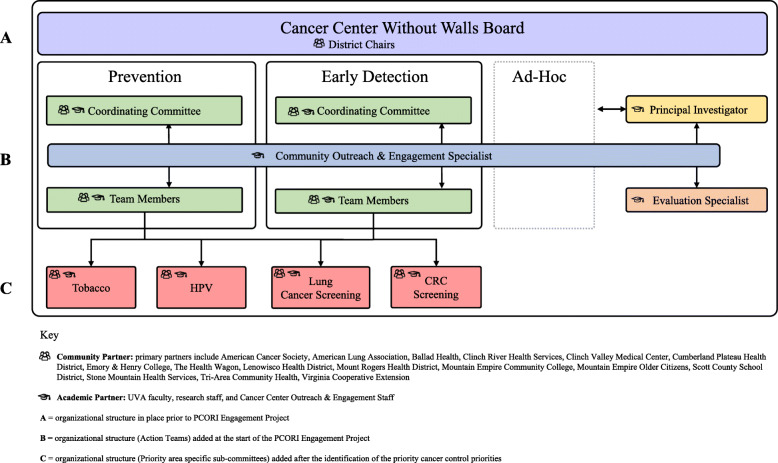
Evolving structure of the Community Advisory Board

At the start of this project, there were 69 (53 community, 16 academic) active CAB members. Approximately two-thirds (65%) were members for a year or more, while about one-third (35%) were newer CAB recruits. CAB members represented community health centers and free clinics, hospitals and health systems, training institutions, and advocacy groups across southwest Virginia. Many members were also cancer survivors and/or caregivers (see Fig. 1). UVA Cancer Center outreach and engagement staff and academic partners from the UVA School of Medicine, Department of Public Health Sciences, and School of Nursing also served on the CAB.

### Meeting structure and the CPPE steps

At the start of this project, CAB members extended the half-day in person meetings from three to four times per year. These meetings consisted of two hours dedicated to sharing updates along with one featured community spotlight and one featured research project. The last two hours of the meetings were dedicated to engaging CAB members in the CPPE process. As the project progressed and priorities were identified, sub-groups began meeting regularly outside of the CAB meetings via conference calls. Meeting decisions informed subsequent activities (e.g., causal model development, strategy rating tasks, training topic focus). A website was developed to share meeting minutes and other resources [37].

The CPPE process is a five step multi-phased and action-oriented approach designed to guide project planning and evaluation. The steps include: (Step 1) problem assessment, (Step 2) identification and selection of potential interventions, (Step 3) planning, (Step 4) setting up a monitoring and evaluation system, and (Step 5) proposal development [10–12]. Each step is flexible with an emphasis on community participation. Given the breadth of cancer disparities and potential cancer control focus areas, the CAB started at Step 1.

### Mixed-methods process evaluation

**Fig. 2:**
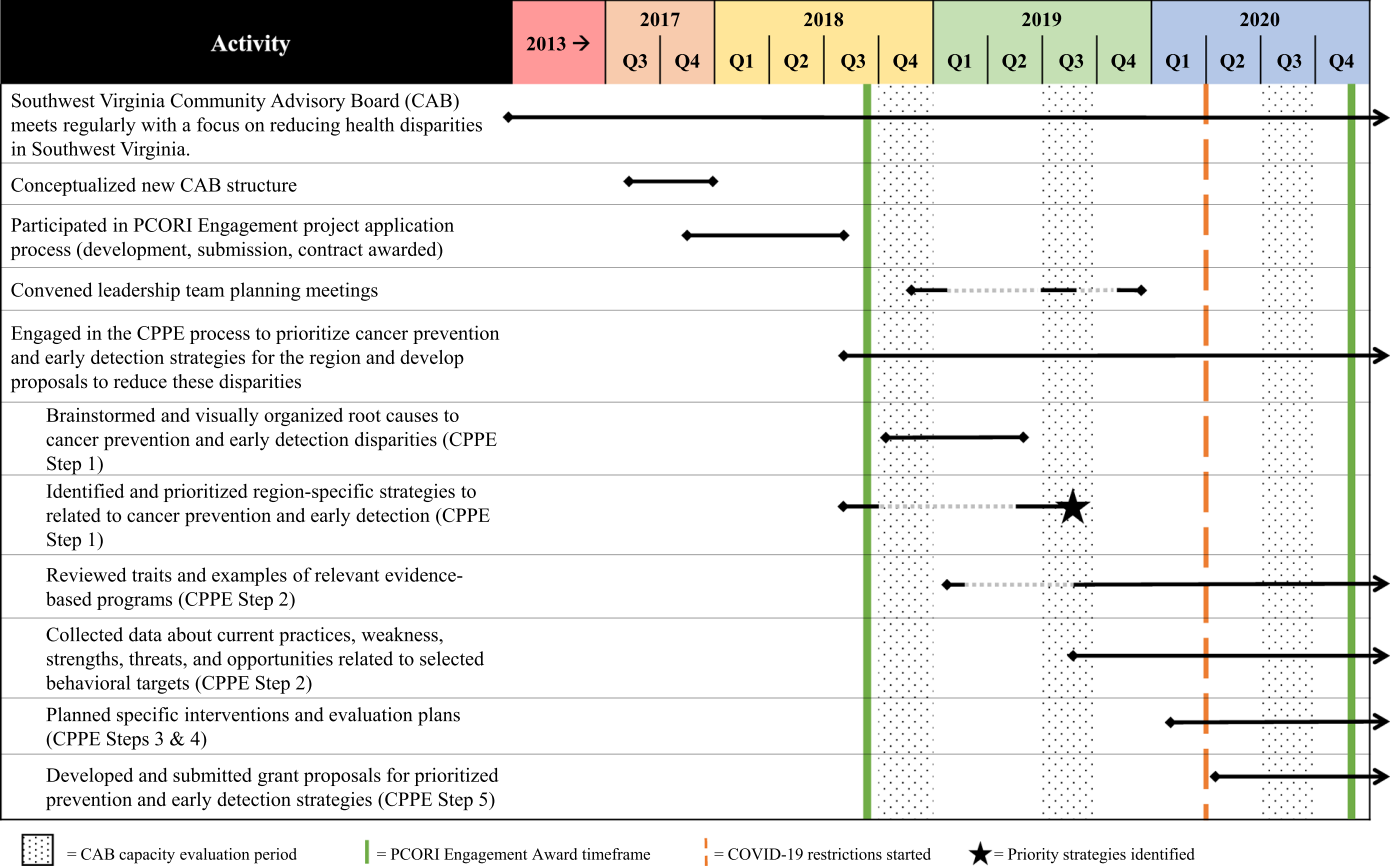
Overview of the capacity building timeline and processes

Quantitative and qualitative data were collected simultaneously, analyzed independently, and triangulated during the data interpretation phase. Over a three-year timespan, CAB meeting agendas, minutes, and meeting artifacts were collected. Additionally, CAB members completed a quantitative survey at three time points (Time 1 = Summer 2018, Time 2 = Summer 2019, Time 3 = Summer 2020) and semi-structured telephone interviews at two time points (Time 2 = Summer 2019, Time 3 = Summer 2020) (see Fig. 2).

The quantitative survey and semi-structured qualitative interview guide were guided by the Nine Habits. The habits were reviewed for relevance to the context of the CAB as well as to the CPPE processes. Seven of the habits were measured, along with trust and satisfaction. Previously developed and validated scales were used to assess each construct [36, 38–41]. Habit descriptions, scale sources, number of items are reported in Table 1. Members completed the survey either at a CAB meeting or via a web-based link. Three trained UVA staff conducted interviews that lasted approximately 30 (range 20–90) minutes.

**Table 1:** Habit descriptions and quantitative scale source and Cronbach’s alpha

*Habit*	*Description*	*Number of items*
**Effective communication**	CAB communication is consistent, purposeful, and uses multiple channels for discussion (e.g., email, Web, live/virtual meetings).	7
**Priority work plans**	Evidence based strategies inform priorities and work plans. Work plans are adaptable and clearly outline outcomes, methods, responsibilities, and timelines that guide CAB efforts.	6
**Clear roles and accountability**	Roles are clearly communicated to CAB members who understand their responsibilities and are accountable for task completion.	4
**Shared decision making**	Shared decision making guides the CAB and procedures are outlined to avoid imbalances in power.	10
**Value-added collaboration**	CAB members acknowledge and appreciate the benefits of collaboration and recognize the power of their collected efforts.	7
**Empowering leadership**	Leaders encourage active participation in decision making by all CAB members. This empowerment builds trust and accountability.	10
**Diversified funding**	Diversified funding can create wider support/involvement in CAB efforts and can secure viability if one funding source disappears.	6
**Trust**	The degree to which CAB members rely on one another to share information, follow through on tasks, and remain committed.	3
**Satisfaction**	Satisfaction with work plan development and execution, goal progress, and allocation of resources.	3

Trust and satisfaction are not habits define by the Comprehensive Cancer Control National Partnership, yet were prioritized and measured constructs for this engagement project. Also, to limit the overall survey and interview length, the habits of dedicated staff and flexible, as defined by the Comprehensive Cancer Control National Partnership, were not measured

### Analysis

Quantitative and qualitative data were first analyzed independently [34, 35, 42]. Quantitative data were analyzed using SPSS. Researchers conducted factorial ANOVAs to explore changes in habit ratings over time by CAB member type (academic or community). To determine potential bias of inclusion of less active members’ data in factorial ANOVAs, the same analysis between time and members type was conducted using a repeated measures ANOVA limited to CAB members who completed the rankings at all three time points.

For qualitative data, interviews were audio-recorded and transcribed verbatim. NVivo 1.2 software was used to manage the hybrid inductive-deductive coding process [43, 44]. Coding took part over four stages, whereby coders maintained memos that identified emerging codes and changes to code definitions [45]. At each coding stage, transcripts were coded independently by two trained researchers and reconciled collectively to resolve discrepancies and refine the codebook. When an agreement could not be reached, a third coder helped resolve discrepancies.

For the first stage of coding, transcripts were segmented into habit constructs. This was followed by the second stage in which coders’ memos during initial segmentation were used to develop codes and definitions reflecting strengths, challenges, and recommendations for each habit. The third stage involved the review of all codes by two researchers to condense overlapping definitions within each habit and across all recommendations. The frequency by which CAB members mentioned each code was calculated to identify prominence of the codes across the two time points [42]. The fourth stage of coding involved member checking and a final reconciliation. Member checking occurred using a summarized qualitative data report, whereby CAB members were asked to review code definitions, counts, and quotes for strengths, challenges, and recommendations [46]. Through this process, 22 members responded to three questions and overwhelming verified that their own experiences were represented and that nothing was missing or misrepresented in the data.

Finally, data triangulation was conducted [34, 35]. The intention was to clarify consistencies and contradictions between meeting artifacts and CAB outputs, as well as distinctions between quantitative and qualitative CAB data strands. This synthesis is presented in the discussion.

## Results

### CAB member participants

Meeting attendance fluctuated, with an average attendance of 28 CAB members, including 16 community and 12 academic members. Of 69 active members at the start of the CPPE process, 49 (71%) signed a consent to participate in the evaluation. At Time 1, 2, and 3, respectively, 45, 32, and 24 CAB members completed the survey, while 37 and 28 CAB members completed the interview at Time 2 and 3, respectively.

### Overall time and activities

Figure 2 provides a timeline of CAB activities and accomplishments. Between June 2017 and September 2020, CAB members engaged in CPPE-related activities to identify and strategize how to address regional cancer priorities. During this time, there were 13 full CAB meetings and two additional training opportunities. Outside of larger meetings, the Prevention and Early Detection Action Teams met an additional four times and, after the four priority areas were identified, sub-groups met six times. A key goal of these meetings and trainings was to increase knowledge exchange pertaining to CCC planning and evidence-based cancer control resources. As such, shared and discussed resources included, but were not limited to the Virginia Cancer Plan [47], Evidence-Based Cancer Control Programs (formerly RTIPs) [48], Community Preventive Service Task Force guide [49], American Cancer Society screening recommendations [50], and Putting Public Health Evidence in Action [51].

Concurrent with engaging in CPPE steps, community and academic CAB members participated in leadership and general capacity building activities. Two in-person, 1-½ day leadership retreats were held at UVA (December 2018, November 2019). Community CAB members and academic leaders reviewed CAB progress and presented to and heard from UVA faculty members whose research was aligned with cancer control priorities. Additionally, a patient-centered outcomes research presentation was held in April 2019 for UVA faculty and a webinar pertaining to social marketing for health behaviors was held for CAB members and other Cancer Action Coalition of Virginia affiliates in February 2020.

### CPPE process preparation and CAB restructuring

CAB members prepared to engage in the CPPE process between June 2017 and April 2018 (Fig. 2). Over four meetings, CAB members reviewed the cancer control continuum and engaged in guided conversations about potential focus, goals, and impacts. CAB members also completed a membership matrix (i.e., organization mission, clientele, staffing, support, allies, interests, future involvement) and brainstormed structures for CAB re-organization. These activities led to a new CAB structure that included Cancer Prevention and Early Detection Actions Team (Fig. 1). These two areas of focus reflected regional cancer data, members’ experience, and organizational missions. The plan included an emphasis on disparities and plans for community and academic member co-leadership. Also, a decision was made to allow ad-hoc sub-groups to address other cancer control priorities, such as survivorship. In anticipation of strategy development during the future CPPE process, CAB members reviewed the Virginia Cancer Plan [47] and data reports highlighting southwest Virginia regional needs specific to prevention and early detection. Collectively, these activities led to the collaborative development of a Eugene Washington PCORI Engagement proposal submission and an awarded contract to support continued CAB engagement.

### CPPE process: steps 1 through 5

As demonstrated in Fig. 2, between November 2018 and September 2019, the CAB participated in three quarterly meetings to engage in problem assessment activities (CPPE Step 1). Activities were facilitated by an external consultant with expertise in CBPR and large group processes. Consistent with CPPE Step 1a (Fig. 2), Action Teams brainstormed root causes for regional cancer prevention and early detection disparities and then developed and refined causal models reflecting root causes (see Supplementary Figs. 1 and 2). Subsequently, Action Teams completed CPPE step 1b (Fig. 2) through a prioritization process to identify the top strategies that could have the most impact on regional cancer disparities. Strategies from the Virginia Cancer Plan [47] were rated on their importance and feasibility. Action Teams then reflected on and discussed ratings in the context of causal models, organizational abilities, and existing regional activities. After several meetings, four priority needs were identified: (1) improving patient and provider education around HPV vaccination, (2) increasing evidence-based tobacco education and behavioral change interventions, (3) increasing advocacy, awareness and screening rates for colorectal cancer (CRC), and (4) increasing advocacy, awareness and screening rates for lung cancer.

CAB members proceeded to CPPE Step 2 through engagement in intervention identification activities. Academic members initiated CPPE Step 2a in February 2019 with a webinar on the science behind evidenced-based programs using examples of cancer prevention and early detection interventions conducted in other rural and/or Appalachian communities (Fig. 2). Then, in Fall of 2019, Action Teams started reviewing evidenced-based interventions related to the CAB’s four targeted strategies. As part of CPPE Step 2b (Fig. 2), this review identified potential individual, system, and social barriers and facilitators to implementing the identified strategies.

Starting in early 2020, sub-groups began to plan specific interventions (CPPE Steps 3 and 4) and submit grant proposals (CPPE Step 5). Due to COVID-19, all of these meetings were conducted virtually. At the time of this paper submission (Winter 2020), the following sub-group progress has occurred: 1) submission of two research grant applications related to HPV vaccination projects, 2) collection of pilot data for a tobacco-based project involving regional pharmacies to develop a research grant application, 3) development of a small media campaign to increase CRC screening rates among four Federally Qualified Health Centers for release during CRC Awareness Month in March 2021, and 4) review of the literature and planning for the potential development and evaluation of a decisional aid related to low-dose computed tomography lung cancer screenings.

### Quantitative capacity findings

**Fig. 3:**
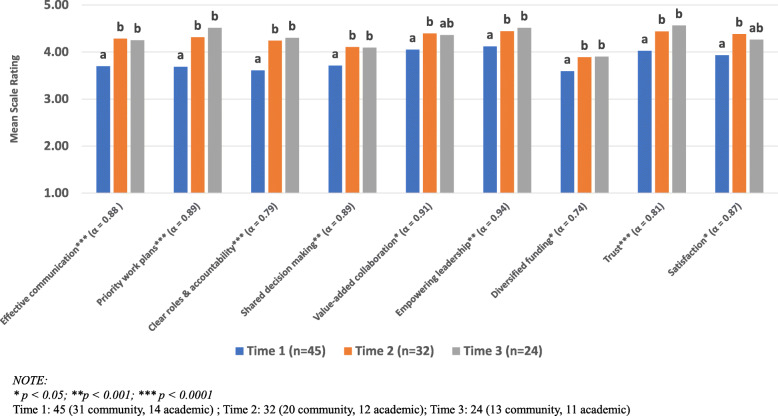
Longitudinal changes in habits of comprehensive cancer control coalitions

Internal reliability for each habit subscale was analyzed using Cronbach alpha. Overall, scale consistencies were deemed acceptable for each habit (Cronbach α = 0.74–0.94; see Fig. 3). As illustrated in Fig. 3, habits were ranked relatively high at Time 1, and all habits significantly improved at Time 2 (all *p* < .05). At Time 3, most habits remained significantly higher than Time 1 (all *p <* .05). Two exceptions were value-added collaboration and satisfaction, which dipped slightly at Time 3 and were no longer significantly different from Time 1 and Time 2. When analyzing data only from CAB members who completed quantitative rankings at each time point (*n* = 16–19 CAB members per habit), findings and interpretations were remarkably similar (data not shown). Likewise, there were no meaningful differences among community and academic members’ ratings of habits (data not shown).

### Qualitative capacity findings

Across habits and at both Time 2 and Time 3 interviews, strengths reported by CAB members outweighed the challenges in both quantity and frequency (Table 2). An exception was for diversified funding, where reported strengths and challenges were relatively more even. Related to challenges, limited time was consistently mentioned across most habits and was viewed as a limiting factor at both time points. Also, implications of COVID-19, especially as it related to effective communication and diversified funding, emerged as a major challenge at Time 3. Many other challenges remained relatively consistent between time points. However, a few challenges decreased from Time 2 to Time 3, including time consuming decision making processes (shared decision making) and bureaucracy of partner organizations (value-added collaboration). Alternatively, a few challenges increased somewhat from Time 2 to Time 3, including difficulty using member expertise and resources (priority work plans), unclear accountability processes (clear roles and accountability), and challenging funding climate (diversified funding).

**Table 2:** Identified challenges and strengths across the habits, Community Advisory Board member counts, and exemplary quotes

Habit	Challenges and strengths	T2^a^	T3^b^	Exemplar quotes
Effective communication	**Challenges**			“I don’t really know that we have made a really solid effort to build awareness outside of our group in terms of what we do and who we are and what we’re about. …it might be in the future that we have a communications action team who could concentrate on that a little bit more.” (Challenge, T2, Academic) “…the elephant in the room is COVID. That…is a huge challenge in terms of how…to facilitate… communication…while being able to…social distance and keep everyone safe.” (Challenge, T2, Community) “I think the leaders do a great job of explaining things and also explaining them more than one time to make sure that you understand. And…create that culture of feel[ing] free to ask if you lack understanding of something.” (Strength, T2, Community) “There’s definitely been a larger push to meeting…more regularly through virtual channels in the last year. And that was…a very good…thing because…there were gaps in between meetings and … recap that was needed…to get everybody back into the swing of things.” (Strength, T3, Academic)
	• Members have limited time to communicate with one another	54%	43%	
	• Lack of strategies for external communication	35%	39%	
	• COVID-19 hindered communication	n/a	43%	
	**Strengths**			
	• Communication uses multiple channels	81%	86%	
	• Communication mirrors the CPPE processes	65%	43%	
	• Communication keeps CAB informed	46%	64%	
	• Communication promotes understanding	43%	46%	
	• Communication is purposeful and keeps momentum	46%	21%	
	• Communication promotes an open and respectful environment	38%	29%	
	• CAB website shares information internally and externally	38%	14%	
	• Communication promotes CAB efforts to the community	32%	32%	
	• Communication from leaders fosters engagement	11%	32%	
Priority work plans	**Challenges**			“… [A]ll the board is volunteer. I think that is a positive and also a challenge. ..[T]hey’re very much committed to participating, but there could be limitations in their time and/or commitment, I guess if they’re pulled in another direction or their organization is. (Challenge, T2, Community) “I think [the CPPE process]…is moving us in a structured environment which we need to be in… For instance, in the last meeting we sat around the table and truly defined what our priorities were and what action steps we would take to implement those different things. [The CAB]… isn’t just a meet and greet and see you again at the next quarter. There’s a lot of action involved and time is well spent.” (Strength, T2, Community) “Well, I like how it’s from the roots up. It’s not you know, we get directed that this is what needs to be done and then we say, well OK, well this is how it works in southwest Virginia. No, we we’re starting from the bottom and working up. And I think, you know, the setting of priorities is coming from the community members.” (Strength, T3, Community)
	• Difficulty using member expertise and resources	11%	36%	
	• Members have limited time to participate in the work plans	14%	25%	
	**Strengths**			
	• Works plans reflect the stage of the CPPE Process	68%	64%	
	• Community stakeholders inform the priorities that drive the work	51%	36%	
	• Priorities are set through shared decision making	41%	25%	
	• Data informs priorities	30%	43%	
	• Work plan roles are informed by member expertise and resources	38%	18%	
	•	22%	36%	
Clear roles and accountability	**Challenges**			“The only thing that I could think of that might be a challenge would be just being able to consistently get the group together like we need to. You know we need to come together as a group as often as we do. And I guess that maybe that could be a challenge just with other people in their responsibilities pulling them in different directions.” (Challenge, T1, Community) “But for the most part people that are in there individuals that are on the CAB that have their certain specialties I think they tend to stay very similar with those roles the roles stay similar.” (Strength, T2, Community) “Well, I think people are asked to do things that either they’ve expressed an interest in or that they may have a good knowledge base or be able to contribute.” (Strength, T3, Academic)
	• Members have limited time to carry out roles	30%	39%	
	• Unclear accountability processes	8%	25%	
	**Strengths**			
	• Accountability is communicated formally and informally	59%	57%	
	• Roles are determined through interest and opportunity	19%	50%	
	• Member knowledge, expertise, and resources influences roles	32%	36%	
	• *(Academic)* Leaders identify roles and members to fill them	22%	36%	
	• Role flexibility and adaptability	27%	32%	
	• Tangible tasks and processes guides roles	30%	29%	
Shared decision making	**Challenges**			“So we have our in-person meetings quarterly and we’ll have these calls in between sometimes. And so, yes, do I sometimes wish things could move a little quicker? It’d be great, but it’s not feasible. It’s not feasible to do that because we make these decisions within the structure that we have and we have the structure for a reason.” (Challenge, T3, Academic) “There is [a formal structure] when we have a decision to make it. If it’s a big decision it is brought to the entire group and then at that point that’s all we have. Both the auditory discussion and the visual things that go up on the board so we can all see what we’re looking at to decide what those priorities are and say okay who votes for this and who votes for that. So there is a formalized process that is taking place there.” (Strength, T2, Community)
	• Decision making process is time consuming	27%	7%	
	**Strengths**			
	• Formal structure for decision-making process	62%	68%	
	• External factors and events integrated into decision processes	49%	54%	
	• Flexibility and adaptability in decision making	51%	32%	
Value-added collaboration	**Challenges**			“And then there’s probably I would think for some of the community members maybe sometimes some tension in like you are a member of the CAB then you’re also from your organization so you’re kind of wearing like multiple hats like you’re sort of part of a bigger organization with a broader mandate but then you’re still like an individual from this particular organization. So trying to balance that might be kind of tricky sometimes.” (Challenge, T2, Academic) “The folks coming there have you know they have resources at their disposal. If within the case of cancer survivors they’re a very valuable resource. And I think you have we have the hospital system there. We have a representative we have home health. We have cancer centers. We have educational institutions. And I think all of them have something to bring to the table. And you know you just to the whole is greater than its parts added paths. So you know I think that’s the value of bringing all those people together.” (Strength, T2, Community)
	• Limited time to devote to collaboration	30%	36%	
	• Narrowed focus may lead to drop out or drop off	30%	29%	
	• Bureaucracy of partner organizations	27%	4%	
	**Strengths**			
	• Pooling resources and expertise of the CAB	62%	93%	
	• Provides learning and networking that is useful and beneficial	62%	54%	
	• Expands reach and influence of the CAB efforts	19%	57%	
	• Provides an inspirational vision	49%	50%	
	• Contributing to research is important	30%	29%	
	• Advancement on the collaboration continuum	3%	29%	
Empowering leadership	**Challenges**			“So my sense is that there’s shared leadership within, you know, the sort of local partners and organizations and on the academic side. And so I definitely have that sense. I mean, I think that’s one thing that’s been really good is that I’ve never had the sense that it’s like UVA’s CAB which would be unfortunate. So it definitely feels like a shared endeavor.” (Strength, T3, Academic) “I think in the same way that they’ve been doing just that by being facilitators and by being leaders and keeping up. … the facilitator has the work laid out for us when we come. … having the work laid out for us so that we know what you know we’ve got we need to get this done to keep this thing moving in the right direction. We’ve got to get this done today or we’ve got to think about this and we’ve got to have a call about this and I’m going to reach out to this or that person and that’s how I see the leadership.” (Strength, T2, Community)
	• Limited time discourages taking on leadership roles	14%	25%	
	**Strengths**			
	• Leaders work to facilitate engagement	51%	61%	
	• CPPE processes open up leadership opportunities	38%	36%	
	• Leadership roles are linked to expertise	24%	36%	
	• Leadership is dispersed and representative of the CAB	27%	25%	
	• Leaders inspire others	22%	25%	
	• Leaders guide, but don’t direct the CAB	16%	25%	
Diversified funding	**Challenges**			“I would like to see more of the budget and the financial breakdown of it. Not just the grant but the entire [CAB] program in general what’s funding it what’s supported and think things like that.” (Challenge, T2, Community) “I think there will be a lot of opportunities. I think right now with Covid that makes it tough because I know a lot of funding is going for that. But I definitely think there’s funding out there for opportunities for obviously cancer related things, especially for rural areas.” (Challenge, T3, Community) “They were pretty upfront with the [budget] especially the PCORI funding. This money was gonna go towards this and this would go towards this. You know how all it was going to work.” (Strength, T2, Community) “Well, of course, all these agencies allowing their people to attend the meetings. That’s a very big in-kind because they could say, well, we’re paying you and you can’t, you know, devote 4 h to that sort of thing. So I think that’s huge right there for those of us that... I don’t have to put in personal time to attend meetings. I think, you know, that’s a biggie.” (Strength, T3, Community)
	• Grant writing is time consuming without guaranteed success	65%	46%	
	• The COVID-19 pandemic has impacted funding opportunities	n/a%	50%	
	• Challenging funding climate	0%	32%	
	• Budget is not transparent	27%	29%	
	**Strengths**			
	• Member expertise in grant writing	78%	64%	
	• Output of CAB used in leveraging grant opportunities	38%	11%	
	• Budget is transparent	32%	29%	
	• In-kind donations by members and organizations	27%	21%	

^a^Time 2: 37 (23 community, 14 academic)

^b^Time 3: 28 (15 community, 13 academic)

As illustrated in Table 2, multiple strengths for each habit were identified at both time points. Similar to challenges, the content and frequency of strengths were fairly consistent from Time 2 to Time 3, with a few notable exceptions. Throughout the CPPE process, CAB members increasingly recognized benefits of the collaboration, including pooling of resources and the collective ability to have greater regional influence (value-added collaboration). Additionally, between Time 2 and Time 3, CAB members more frequently expressed opportunity for roles for which they were passionate (clear roles and accountability) and trusted leadership to identify roles and guide CAB efforts (empowering leadership). Furthermore, there was increased frequency of identifying how effective communication kept members informed and fostered engagement. Alternatively, at Time 3, CAB members less frequently mentioned purposeful communication and the website as strengths (effective communication). There were also decreases in frequency of perceptions concerning the CAB’s leveraging of community partner expertise to inform priorities and work plans (priority work plans), flexibility around decision making (shared decision making), and ability to obtain grant funding (diversified funding) between Time 2 and Time 3.

As shown in Table 3, interviews identified nine key recommendations that cut across the habits. Recommendations reflect opportunities to promote multiple stakeholder perspectives, recruit new members, seek diverse funding sources, improve internal and external communication, develop sustainability plans, identify and leverage resources, expand leadership, and enhance ownership of CAB efforts. Overall, CAB members mentioned most recommendations at a relatively consistent frequency at both time points. However, relative to Time 2, at Time 3 CAB members more frequently recommended efforts to improve external communication strategies, expand pathways to leadership roles, and enhance ownership of CAB efforts.

**Table 3:** Key recommendations to promote capacity for regional, rural comprehensive cancer control across the habits

Recommendations	Associated Habits							Time 2% (of 37)	Time 3% (of 28)
	*Effective communi-cation*	*Priority work plans*	*Clear roles/ account-ability*	*Shared decision making*	*Value-added collaboration*	*Empowering leadership*	*Diversified funding*		
1. Use clear communication strategies to foster clarity and unified understanding within the CAB	✓		✓	✓			✓	65%	75%
2. Improve external communication strategies to promote CAB efforts	✓							43%	64%
3. Strategically recruit new members for mission enhancement					✓	✓		38%	32%
4. Seek diverse funding to sustain CAB operations/work plan projects							✓	35%	39%
5. Promote an open, equitable space for multiple perspectives			✓	✓		✓	✓	33%	25%
6. Develop formal processes to support CAB sustainability (e.g., funding capacity, accountability structures, collaborative action planning)		✓	✓		✓		✓	24%	29%
7. Expand pathways for members to obtain leadership roles and/or positions			✓			✓		14%	25%
8. Enhance perceptions of ownership in CAB efforts by CAB members and their respective organizations			✓		✓			11%	29%
9. Identify, secure, and leverage existing and needed CAB resources	✓		✓		✓	✓		11%	18%

## Discussion

Advancing, managing, and sustaining CCC coalition efforts is not an organic process. Rather, it takes concerted efforts and thoughtful, purposeful partnership building approaches [13]. Despite steady US progress pertaining to CCC efforts over the past two decades [18, 19], evidence suggests that rural communities are lagging behind [15–17]. Thus, there is a clear need to link cancer control outreach and engagement efforts with research efforts. Numerous studies document the promise of CBPR approaches in achieving this link. Examples include the value of community involvement in promoting high intervention ownership and considering organizational capacity [52], the necessity of equalizing power distribution within diverse partnerships [53], and the importance of building multi-sector coalitions to strengthen capacity and promote community resiliency [54]. This case study is unique in that we draw from a key community asset (e.g., a functioning CAB with established multi-sector collaboration and history of advising cancer outreach and research projects) and build from prior and on-going successful projects [37, 55–57]. Established coalition building tools were used to work collaboratively with the CAB to pivot its structure from opportunistic partnering to action oriented planning thereby strengthening its function to address cancer related disparities. The robust process evaluation and mixed-methods approach provided an in-depth and rich reflection of opportunities and challenges in redirecting coalition efforts and advancing community CAB members along the engagement spectrum from informing to stakeholder directed research project [7]. In sum, this case study responds to literature gaps concerning useful processes that can actively engage community members in formulating and implementing CCC research agendas and outreach projects.

The outcomes of our CBPR approach and CPPE processes indicate CAB success in identifying priority CCC issues, co-developing research questions, and transitioning into action-oriented intervention testing and implementation in one rural, medically underserved region. Specifically, efforts resulted in the reorganization of the CAB to address key regional cancer needs around prevention and early detection and the building of causal models to identify strategies to address those needs and reduce cancer disparities within the region. These processes spurred collaborative efforts to develop public relation and clinical trial research opportunities surrounding HPV vaccination awareness, tobacco education and cessation programming, and screening for CRC and lung cancer. Furthermore, evaluation of effective CCC habits was key to providing insight on the internal functioning of the CAB and its readiness to proceed to implementation phases of the CPPE process (e.g., Step 5).

CCC initiatives can be overwhelming, particularly in rural, health disparate regions where epidemiological data and social determinants of health indicators suggest that nearly every cancer control strategy is a priority. Studies demonstrate that narrowing priorities in these situations are difficult [58]. In our case, the Virginia Cancer Plan has 12 goals that include about 46 objectives and 155 strategies [59]. While the list is comprehensive, addressing each objective is not possible for a regional cancer coalition operating in a medically underserved region. Consistent with these observations, the CAB readily synthesized data into comprehensive causal models in Step 1 of the CPPE process, but the breadth and depth of regional cancer disparities made prioritization of strategies in Step 2 considerably more difficult. As an example, the Prevention and Early Detection Action Teams struggled to narrow five top rated priorities down to two. To help alleviate this struggle, the CPPE facilitator consistently reminded the CAB of the following: “*Saying no right now, doesn’t mean saying no forever;*” “*We can’t take on everything and do it all well;*” and “*We need to concentrate on where high priority in your communities and within your organizations overlap with the potential for high impact*.” After about six meetings, our CAB eventually landed on a set of four focused CCC priorities, two within each action team.

Evaluation of CCC coalition best practices revealed key insights into strengths and challenges of our CAB. Prior to this engagement project in 2017, the CAB existed for about 4 years, during which time they developed a strong sense of commitment and collaboration. This is reflected in the high quantitative habit ratings at Time 1. These ratings signify the strong foundations of the partnership and are consistent with the CAB’s yearlong restructuring and preparation for an action-oriented agenda. Still, longitudinal evaluation demonstrated continued quantitative improvements in rated habits. Qualitative data, in large part, supported these improvements and suggested that they may be attributed to CPPE and CBPR efforts along with guidance from the National Comprehensive Cancer Control Program’s Nine Habits resource.

Positivity of the quantitative data was reflected in CAB member emphasis on strengths over challenges during the interviews. Moreover, qualitative data suggested an increased sense of efficacy in the CAB’s collective abilities as they moved forward in carrying out work plans. Although some identified strengths at Time 2 fluctuated at Time 3, this variation is linked more to changing circumstances surrounding the progression through the CPPE process, the approaching end of current funding, and the COVID-19 pandemic than to reduced or changing capacity of the CAB. For instance, the time-consuming process of deciding on priorities was in progress during Time 2 interviews and completed by Time 3, reflecting a decrease in challenges mentioned around timeliness of this activity. Similarly, with evolution from priority setting to action planning, emphasis shifted to a need for identifying and utilizing member expertise and resources, thus making this a greater challenge at Time 3 than at Time 2. Uncertainty as the CAB moved forward were demonstrated as some previously mentioned assets waivered, and challenges increased around the diversified funding habit. Due to COVID-19, anxiety was likely heightened because of changes to the funding landscape and to shifting priorities of members and their organizations to deal with a health crisis that may limit their availability to participate in CAB activities [60]. However, despite some uncertainty around transitions in the CPPE process and in potential future funding sources, the CAB was seen as both a solid and a powerful force for change that benefited from diverse representation, committed and passionate members, and experienced leaders. This is the backbone of the CAB that will sustain efforts as they navigate into the next CPPE phase and obtain funding to carry out sub-group work plans.

### Ongoing and next steps

While still somewhat early in the planning, evaluation and proposal development CPPE steps, three examples signify community CAB members’ progression along the engagement spectrum to collaborating and directing projects. First, the HPV sub-group has submitted two research proposals focused on multi-sector capacity building as well as selecting and pilot testing vaccination interventions. Second, one Federally Qualified Health Center partner has led submission of a Health Resources and Services Administration Rural Health Care Services Outreach Program Grant that focused on cancer control. Finally, one of our ad hoc sub-groups has submitted an American Cancer Society research grant to evaluate a cancer survivorship intervention tailored to the needs of rural patients and health systems. Each project focuses on intervening on regional cancer disparities and is guided by evidence-based resources.

After both data collection time points, a summary of key recommendations was shared and discussed at CAB meetings. Efforts to better coordinate internal and external communication were prioritized first [61]. Enacted strategies include development of a website to disseminate CAB information, launch of a monthly newsletter emailed to CAB members, coordinated and standing teleconference sub-group meetings, and launch of a local newspaper series to promote external CAB visibility and to raise awareness about pertinent cancer priorities. As the CAB continues its sub-group structure transition, recruiting new members and perspectives has emerged as the next priority, especially since the sub-group structure may stretch members too thin and negatively impact participation. Similarly, promoting a shared leadership structure within the sub-groups and stimulating communication within and across sub-groups is imperative to our continued efforts.

A future challenge and opportunity for the UVA Cancer Center is to remain accountable to the CAB and CPPE process. This involves continuing to infuse resources, such as regional pilot projects and technical assistance to support the CAB’s function, as well as bringing research content and CBPR expertise to the table to match community-identified priorities and leverage external resources.

Although the PCORI engagement contract has ended, the action-oriented processes will continue. As stated by Pyron and colleagues, the interaction of a CCC program can be summed up with three Ps: *partnerships*, CCC *plans*, and CCC *program* interventions [21]. While our CAB has made great strides by harmonizing partnerships and focusing priority plans, we are in the infancy of planning and implementing CCC program interventions. The CPPE framework continues to serve as our framework as we plan projects and develop proposals. As the CAB sub-group efforts advance, we continue to rely on evidence-based resources [48–51]. Likewise, as we strategizes on real-world application and sustainability of efforts in practice-based settings across rural Appalachia, participatory implementation science is at the forefront of our agenda [62].

### Limitations

Primary limitations of our case study is the relatively small sample and potential lack of generalizability. While we were able to detect statistically significant changes in the habits over time, limited statistical power may have impacted lack of significant difference detected between community and academic members. Also, our CCC project is embedded within a unique set of contextual factors in rural Appalachia and the identified challenges, strengths, and recommendations are unique to our circumstances. Moreover, we provide frequency counts to help triangulate quantitative and qualitative findings and to explore shifts in CAB perspectives over time [42]; however, there are some limitations on drawing meaning from frequency counts. Finally, we relied on the Nine Habits to help conceptually guide our process, yet decided to use other validated scales with high psychometric properties instead of those offered in the resource guide [13, 14]. Despite these limitations, our study also has several strengths. Examples include the mixed-methods study design, application of a strong conceptual framework, use of an external facilitator to guide group processes, number and diversity of interviewed participants, member checking to verify findings, and robust hybrid inductive-deductive qualitative analysis. Other program planners and researchers may be able to apply our methodologies to advance priority setting and capacity building in their own coalition advancement efforts.

## Conclusions

This engagement project exemplifies an equity-based participatory approach aimed at setting and advancing a research agenda to address persistent gaps in rural cancer control in Appalachia. The CBPR approach used a CPPE process in conjunction with internal evaluation of CCC coalition best practices to advance CAB efforts. We intend to actively engage the CAB in priority identification and capacity development and are optimistic that doing so will enhance the likelihood that they will remain highly involved in CCC projects. Continued efforts to augment CAB identified strengths, attend to specific challenges, and enact concrete tactics to address emergent recommendations will be key to the CAB’s sustained engagement and future impact on regional cancer disparities. These engagement processes may serve as a template for similar coalitions in rural, underserved areas. The demand for action-oriented partnership development and need to translate evidence-based CCC research into practice is especially high among disadvantaged, rural regions that face countless cancer control and social determinants of health inequities. CPPE process may be an effective means for guiding these coalitions to action. Likewise, strong coalitions are sustained through buy-in, meaning members participate in and are vested in planning, implementation, and evaluation [21, 63]. Evaluating coalitions for effective practices is a means to identify strengths, continued challenges, and opportunities that promote discussion and action around strengthening members’ commitment as well as coalition structure and processes.

## Supplementary Information

**Additional file 1: Supplementary Fig. 1.** Causal models from Prevention Action Team.

**Additional file 2: Supplementary Fig. 2.** Causal models from Early Detection Action Team.

**Additional file 3: Supplementary Table 1.** GRIPP2 Short Form

## Data Availability

Not applicable.

## Abbreviations

CAB: Community-Academic Advisory Board
CBPR: Community-based participatory research
CPPE: Comprehensive Participatory Planning and Evaluation
CCC: Comprehensive cancer control
CDC: Centers for Disease Control and Prevention’s
NCCCP: National Comprehensive Cancer Control Program
NCI: National Cancer Institute’s
NIH: National Institutes of Health
PCORI: Patient-Centered Outcomes Research Institute
US: United States
UVA: University of Virginia
HPV: Human papillomavirus
CRC: Colorectal cancer
