# Supplementary material for: Advancing engagement and capacity for rural cancer control: a mixed-methods case study of a Community-Academic Advisory Board in the Appalachia region of Southwest Virginia
Source: Res Involv Engagem. 2021 Jun 22;7:44. doi: 10.1186/s40900-021-00285-y (PMC8218281; doi:10.1186/s40900-021-00285-y)
Supplement: Supplementary file 1 — Additional file 1: Supplementary Fig. 1. Causal models from Prevention Action Team. [file 40900_2021_285_MOESM1_ESM.pdf]

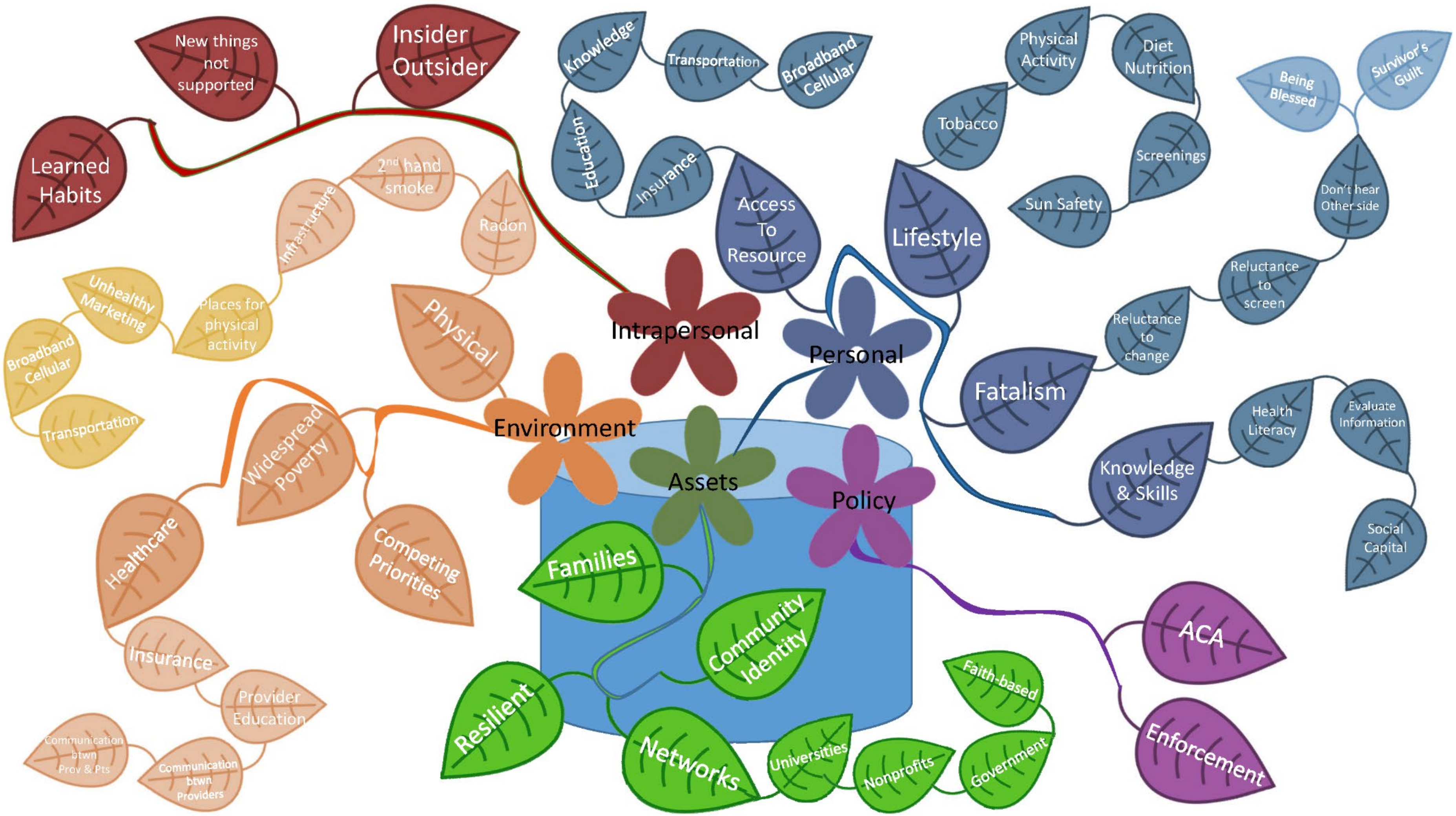

Factors that influence Cancer Prevention:

- Environmental Factors
- Intrapersonal Factors
- Personal Factors
- Policy Factors
- Assets (all levels) Factors
