# Supplementary figures and images for: Advancing engagement and capacity for rural cancer control: a mixed-methods case study of a Community-Academic Advisory Board in the Appalachia region of Southwest Virginia
Source: Res Involv Engagem. 2021 Jun 22;7:44. doi: 10.1186/s40900-021-00285-y (PMC8218281; doi:10.1186/s40900-021-00285-y)

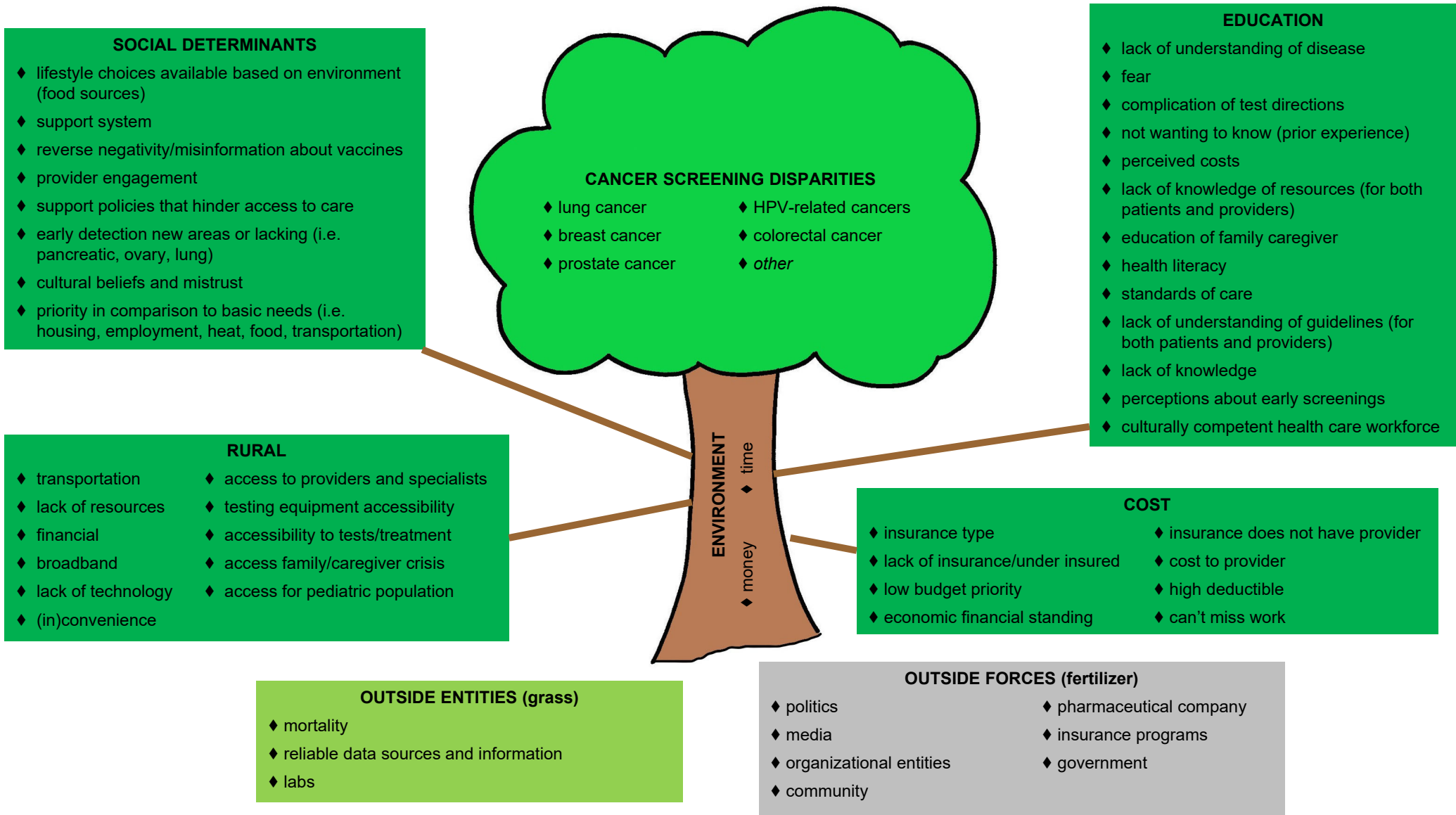

Supplement: Supplementary file 2 — Additional file 2: Supplementary Fig. 2. Causal models from Early Detection Action Team. [file 40900_2021_285_MOESM2_ESM.pdf]
