# Supplementary material for: Advancing engagement and capacity for rural cancer control: a mixed-methods case study of a Community-Academic Advisory Board in the Appalachia region of Southwest Virginia
Source: Res Involv Engagem. 2021 Jun 22;7:44. doi: 10.1186/s40900-021-00285-y (PMC8218281; doi:10.1186/s40900-021-00285-y)
Supplement: Supplementary file 3 — Additional file 3: Supplementary Table 1. GRIPP2 Short Form [file 40900_2021_285_MOESM3_ESM.docx]

**Supplementary Table 1.** GRIPP2 Short Form

| Section and topic | Item | page No |
| --- | --- | --- |
| 1: Aim | Report the aim of PPI in the study | pp. 7-8 |
| 2: Methods | Provide a clear description of methods used for PPI in the study | pp. 8-9 |
| 3: Study Results | Outcomes—Report the results of PPI in the study, including both positive and negative outcomes | pp. 10-14 |
| 4: Discussion and conclusions | Outcomes—Comment on the extent to which PPI influenced the study overall. Describe positive and negative effects | pp. 14-16 |
| 5: Reflections/critical perspective | Comment critically on the study, reflecting on the things that went well and those that did not, so others can learn from this experience | pp. 17-19 |
